# Creative Adaptability: Conceptual Framework, Measurement, and Outcomes in Times of Crisis

**DOI:** 10.3389/fpsyg.2020.588172

**Published:** 2021-01-12

**Authors:** Hod Orkibi

**Affiliations:** Faculty of Social Welfare and Health Sciences, University of Haifa, Haifa, Israel

**Keywords:** creativity, adaptation, adaptability, well-being, stress, COVID-19, Corona virus, psychodrama

## Abstract

This article presents the framework and explores the measurement, correlates, and outcomes of creative adaptability (CA), proposed here as the cognitive–behavioral-emotional ability to respond creatively and adaptively to stressful situations. Data collection was in April 2020, during the peak of the outbreak of the Coronavirus pandemic (COVID-19) in Israel. In Study 1, a sample of 310 adults completed the newly developed CA scale, as well as spontaneity, openness to experience, creative self-efficacy, and well-being measurements. The results of exploratory and confirmatory factor analyses corroborated the 9-item CA scale’s theorized underlying construct. The scale’s validity and reliability were also supported. Exploratory analyses suggested that the association between CA and well-being was mediated by creative self-efficacy and that CA may buffer the impact of individuals’ concern about Coronavirus on their well-being. In Study 2, short-term longitudinal data based on a sample of 71 students suggested that CA may predict lower psychological stress over time. Support for the CA scale’s internal consistency reliability was obtained and its test–retest reliability was established. Overall, the results shed light on this new construct as a potential protective factor. Implications for theory, research, and practice are discussed.

## Introduction

In times of crisis such as the COVID-19 pandemic, people’s personal protective factors are highly consequential to their well-being (Chen and Bonanno, 2020). Creativity as a personal protective factor has generally received little attention compared to studies on the creativity–psychopathology association and studies on finalized creative outcomes such as ideas (creative thinking), solutions (problem solving), or products (scientific, artistic), possibly due to the fact that such measurable outcomes lend themselves more readily to empirical investigations (Kaufman, 2017). The purpose of this article is to introduce *creative adaptability* (CA), defined here as the personal ability to generate new and effective cognitive–behavioral–emotional responses to stressful situations. The discussion of the CA framework and measurement is followed by data collected in two studies on adults, during the peak of the outbreak of the Coronavirus pandemic in Israel. The results shed light on this new construct as a potential protective factor, informing not only theory and research but also directions for practice.

## Creativity and Well-Being

Whereas the link between creativity and mental illness has been studied for decades (Kaufman, 2014; Thys et al., 2014; Martín-Brufau and Corbalán, 2016), humanistic scholars and positive psychologists have long argued for a link between creativity and well-being indicators such as adjustment, optimal functioning, and health. For example, J. L. Moreno, the founder of psychodrama, suggested that creativity is essential for adapting to life changes and unexpected challenges (Moreno and Moreno, 1944). He posited that “people must be creative in order to survive” (Moreno, 1964, p. 158) and that for creativity to emerge it needs to be catalyzed by spontaneity (Moreno, 1955, p. 365). Moreno defined spontaneity as a pro-creative catalyzing state of readiness that propels “the individual toward an *adequate* response to a new situation or a *new* response to an old situation” (Moreno, 1953/1993, p. 42, emphases added). The adequacy of a response is a function of its suitability to the requirements of a given situation (Moreno, 1972/1994, p. 93), whereas the newness of a response refers to being “fresh, novel, creative, in the here and now, not fore-ordained or predetermined, but arising out of the immediate situation…” (Moreno, 1969, p. 213). Carl Rogers (1961) viewed creativity as an underlying motivational force for growth, and creative behaviors as satisfying and self-actualizing. Maslow (1962) believed that creativity facilitates self-actualization and is related to greater spontaneity, self-acceptance, unity, integration, and synergy within a person. Positive psychologists have classified creativity as cognitive character strengths under the virtues of wisdom and knowledge (Peterson and Seligman, 2004) and have specifically referred to creativity as the ability to generate ideas or behaviors that are recognizably original, novel, surprising, or unusual, as well as adaptive.

Studies have shown that creative tendencies, measured as openness to experience, correlate negatively with mortality and poor health (Ferguson and Bibby, 2012; Turiano et al., 2012) and that creative self-efficacy (i.e., an individual’s self-belief in his/her ability to be creative when required by a situation; Tierney and Farmer, 2002) correlates positively with adaptive posttraumatic growth and mental health (Orkibi and Ram-Vlasov, 2019). In his extensive discussion of creativity and health, Runco (2007) concluded that creativity is associated with adaptability, which he termed “one of the most powerful concepts in the creativity literature” (p. 161). There is growing evidence that high creativity is related to low stress and to the capacity to adapt and cope (Runco, 2014). Simonton (2002) used the term “adaptive originality” to define creativity, and Cropley (2006) stated that without being “adapted to reality” creativity is “only quasicreativity or pseudocreativity” (p. 391). Overall, there are consistent indications of a link between creativity, adaptability, and well-being.

## Creative Adaptability

The bipartite standard definition of creativity suggests that creativity is the ability to generate outcomes that are both new (or original, unusual, unique) and effective (or adaptive, appropriate, suitable, useful) (Runco and Jaeger, 2012). However, *new* and *effective* are relative concepts. Whereas creative ideas, solutions, or products are often judged as new and effective by experts in a given domain (Kaufman and Baer, 2012), who is best suited to judge the newness and effectiveness of personal psychological “products” such as cognitive, behavioral, and emotional responses?

The answer to *new* seems straightforward: new, rather than routine or habitual, is defined from the individual’s perspective. That is, something is considered new from an internal frame of reference in comparison to the individual’s own past experiences in context. This idea echoes Runco’s (1996, 2011) theory of personal creativity, where creativity does not necessarily require an external frame of reference. Rather, “it relies on the individual’s own personal logic, with personal criteria for the usefulness and originality of a solution” (Runco, 1996, p. 5). A response can be considered as *effective* if it maximizes positive outcomes and minimizes negative outcomes. However, Averill (2005) stressed that because effectiveness is a relative concept, a response that is effective for one individual may be ineffective or even deleterious for the group and vice versa. Furthermore, a response that is considered effective or beneficial in the short term may eventually prove ineffective or harmful in the long term and vice versa. Thus, the effectiveness of a response may sometimes be subject to retrospective reevaluation because it may depend as much on hindsight as on foresight (Averill et al., 2001; Averill, 2005). This view coincides with Smith and Smith’s (2017) “1.5” criterion model of creativity, where novelty is the “1” criterion and “0.5” indicates that to be considered creative, an idea only needs to have the *potential* to be useful or effective (hence 0.5 and not 1), rather than having already demonstrated its usefulness of effectiveness. When applied to CA, the 0.5 criterion suggests that a response, at minimum, should have the potential to be effective, namely, the likelihood of being beneficial for individuals who experience stress in a given context.

Creative efforts are often motivated by the perceived need to solve a problem or deal with tension and stress. Runco (1999) noted that the perceived need for adaptation depends on *subjective* personal interpretations of experiences, which may motivate a person to adopt new cognitions, emotions, and behaviors. For example, the COVID-19 pandemic has forced teachers worldwide to adapt to online teaching (König et al., 2020; Moorhouse, 2020; Scull et al., 2020). However, whereas one teacher may interpret this new situation as a threatening and stressful challenge and may stick to tried and true teaching methods that are however inappropriate for online teaching, another teacher may perceive this challenge as an exciting opportunity to embrace innovative methods to creatively adapt to the new situation. In addition, tension and stress may arise although there is little or no input from the environment. This can take the form of intrapersonal tension between logical inferences and emotions, between contradicting emotions, or between imaginative ideas and evidence (Runco, 1999). Moreover, the need to adapt may arise proactively, in response to perceived intrapersonal tension, and/or reactively, in response to perceived external–environmental tension or stimuli. That is, the source of the tension, disparity, or disequilibrium can be internal and/or external to the person. This notion is consistent with theories on individual adaptability, where adaptability is seen as an individual difference that varies across people and influences how they interpret and behave across situations (Ployhart and Bliese, 2006).

Importantly, creativity and adaptability are related but distinct constructs. One key difference between the two is that creativity necessitates novelty, unconventionality, and non-conformity, whereas in some cases conformity and compliance are the optimal adaptable response (Runco, 1999). Thus, adaptability may sometimes preclude creativity (Cohen, 2012). Creativity can also be maladaptive when it brings about destructive consequences to the self and others. This is often referred to as “the dark side of creativity” or “malevolent creativity” that may involve criminal behavior (Cropley et al., 2010). Likewise, poor adaptability in the form of the “crazy artist” and “mad genius” stereotypes suggest a link between extreme creativity and psychopathology (Simonton, 2014).

Both creativity and adaptability are often related to psychological flexibility, which involves the ability to adapt to various situational demands and reconfigure mental resources (Kashdan and Rottenberg, 2010). Torrance (1995) defined creativity as the ability to think in an original and flexible way to solve problems and adapt to real-life situations. Cohen (2012) suggested that mature creativity may enable adaptation through internal transformation of the self. This internal transformation requires sensitivity to one’s self, openness, and willingness to modify the present experience, effortful and active adaptation of perceptions, and tolerance of uncertainty or ambiguity. Attempts to make sense of an uncertain situation may catalyze creative thoughts and actions because “uncertainty typically requires us to challenge our old assumptions and try new things” (Beghetto, 2019, p. 33).

It is worth underscoring the two ways in which CA differs conceptually from the two related constructs of resilience and coping. First, CA, by definition, involves the ability to generate a novel response, whereas resilience does not; rather, resilience involves the ability to bounce back (or recover) from adversity in terms of a manifested return to a previous (rather than new) level of functioning and performance (Windle, 2011). Second, CA is situated within the context of a changed or stressful situation, whereas resilience is usually situated within the context of exposure to trauma, significant threat, or severe adversity (Luthar and Cicchetti, 2000; Luthar et al., 2000). CA differs conceptually from coping in that the former is defined by the potential to generate an adaptive response. Conversely, some conceptualizations of coping also consist of responses that are potentially dysfunctional or maladaptive, such as disengagement coping strategies that involve avoidance, denial, and passive wishful thinking, which are generally not effective in reducing distress in the long run (Carver and Vargas, 2011).

Thus, the construct of *creative adaptability* (CA) presented here refers to one’s ability to respond creatively and adaptively to stressful situations. More specifically, CA involves the ability to generate personally new and effective cognitive, behavioral, and emotional responses to stressful situations that may lead to positive outcomes (hence the “1.5” criterion). Cognitive CA refers to generating personally new and potentially effective ideas, perspectives, and thoughts; behavioral CA refers to executing personally new and potentially effective behaviors and actions; and emotional CA refers to generating personally new and potentially effective emotional reactions. Altogether, as in cognitive–behavioral therapy (Beck, 2011; Azoulay and Orkibi, 2015), these three dimensions are theorized to be interrelated and malleable and are therefore pertinent not only to theory and research but also for practice, as suggested in section “General Discussion.”

## Study 1

Study 1 explored the measurement and correlates of CA of adults in the stressful situation of the COVID-19 outbreak. Based on the framework discussed above, the following were hypothesized:

Hypothesis 1: CA will positively correlate with CSE, openness to experience, and spontaneity.

Hypothesis 2: CA will positively correlate with well-being.

Hypothesis 3: the association between CA and well-being will be mediated by creative self-efficacy (CSE).

Hypothesis 4: CA will moderate the negative association between concern about Coronavirus and well-being.

### Materials and Methods

#### Sample and Procedure

In April 2020, a sample of 310 adults was recruited from an online panel service, during the peak of the outbreak of the Coronavirus pandemic (COVID-19) in Israel.

Because of prolonged countrywide lockdown orders, many people were worried about purchasing enough food and medication, and many suffered financial losses and experienced distress (Pfefferbaum and North, 2020; Shapiro et al., 2020). The sample was composed of 51% females, aged 18–84 (*M* = 41.6, *SD* = 16.38), of whom 85% were born in Israel and the rest in “other” countries. Of the total sample, 39% lived in the center of Israel, 26% in the north, 19% in the south, 8% in Jerusalem area, and 8% in the Sharon coastal plain area; 98% were Jewish, 52% were secular, 31% traditional, 14% observant, and 3% ultra-orthodox. In the sample, 66% were married or living a partner, 26% were single, 6% were divorced or separated, and 2% were widowed. Most participants (61%) had children, and 62% reported that their financial situation pre-COVID was “average,” 21% reported “below average,” and 17% reported “above average.” Most participants (32%) had a bachelor’s degree, 25% had post-high school vocational training, 22% had a high school diploma, 18% had a master’s degree, and 3% had a PhD.

This study was approved by the Ethics Committee for Human Experiments at the University of __ (approval no. 397/16, as part of a larger research project). Logging in to the survey platform (Qualtrics) indicated consent and completion took about 20 min. Since all questions were mandatory, there were no missing data.

#### Measures

##### Creative adaptability

The CA scale was developed to capture participants’ cognitive–behavioral–emotional abilities to respond creatively and adaptively to stressful situations. The initial version included 15 items, but exploratory and confirmatory factor analyses yielded a final 9-item version, as described in the Results section below. Participants were asked: “please indicate to what extent each of the following statements describes how you *usually* are in stressful situations.” Items were rated on a 5-point scale from 1 (*not at all like me*) to 5 (*very much like me*).

##### Openness to experience

Trait creativity was measured on the 10-item openness to experience subscale of the Big-5 questionnaire (John et al., 2008). Participants were asked to indicate the extent to which they agreed with each of the statements about how they see themselves, on a scale from 1 (*strongly disagree*) to 5 (*strongly agree*). A sample item is: “I see myself as someone who values artistic, aesthetic experiences.”

##### Creative self-efficacy

CSE was measured on the 6-item creative self-efficacy scale (Karwowski, 2016). Participants were asked to indicate the extent to which they agreed with each of the statements on a scale from 1 (*strongly disagree*) to 5 (*strongly agree*). A sample item is: “I know I can efficiently solve even complicated problems.”

##### Spontaneity

The tendency to be spontaneous was measured on a 5-item subscale from a larger playfulness scale (Shen et al., 2014). Participants were asked: “Please indicate to what extent each of the following statements describes how you usually are.” Items were rated on a scale from 1 (*not at all like me*) to 5 (*very much like me*). A sample item is: “I often do unplanned things.”

##### Well-being

The World Health Organization’s 5-item Well-Being Index (WHO-5) examines respondents’ health condition in the last month, with higher scores indicating greater well-being (Topp et al., 2015). Items are rated on a scale from 1 (*never*) to 5 (*always*). A sample item is: “I woke up feeling fresh and rested.”

##### Demographics and background

Data were collected on age, gender, religion, religiousness level, area of residence, marital status, children, education level, and financial status. The participants were asked “To what extent have you been personally affected by the Coronavirus pandemic?” and “How concerned are you about the Coronavirus pandemic?” which were rated on a scale from 1 (*not at all*) to 5 (*a great deal*).

#### Data Analysis

The preliminary analyses consisted of an exploratory factor analysis (FFA) and a confirmatory factor analysis (CFA) of the CA scale. First, to increase general validity and to avoid overfitting of the measurement model, the *N* = 310 sample was randomly split into two groups using SPSS. v25, such that 50% of the participants were randomly selected (*n* = 155) for the computation sample (EFA training dataset) and the remaining participants (*n* = 155) were placed in the cross-validation sample (CFA test dataset). To robustly explore the factor structure of the initial 15 items, several criteria were considered to determine the number of factors (Pituch and Stevens, 2016). These included factors that (1) had eigenvalues greater than 1, (2) were suggested by inspecting a scree plot of eigenvalues, (3) had eigenvalues larger than expected by chance as obtained by parallel analysis (Patil et al., 2008, 2017), and (4) were conceptually coherent.

To assess the measurement model and construct validity, a CFA was performed using Amos v25. Model fit to the data was evaluated using the criteria of χ^2^/df ≤ 3, comparative fit index (CFI) ≥ 0.95, Tucker–Lewis coefficient (TLI) ≥ 0.95, root mean square error of approximation (RMSEA) ≤ 0.08, and standardized root mean square residual (SRMR) ≤ 0.08 (Schreiber et al., 2006).

These indices were also used in structural equation modeling (SEM) to test the theorized mediation model for the association between CA as a latent variable and well-being. A robust bootstrap method for testing indirect effects (i.e., mediation) was employed with the confidence level set at 0.95 and bootstrap bias-corrected samples set at 1,000. When zero is not within the 95% confidence interval (CI), the indirect effect is significantly different from zero at *p* < 0.05, two-tailed (Preacher and Hayes, 2004, p. 722). Hayes’ (2018) SPSS PROCESS macro v3.4 (Model 1) was used to tested the moderation model.

### Study 1 Results

#### Preliminary Analyses

##### Exploratory factor analysis

A statistically significant Bartlett’s test, *χ*^2^ = 1506.950, *df* = 105, *p* < 0.001, and a Kaiser–Meyer–Olkin statistic (KMO = 0.902) greater than its threshold value of.50 indicated that the data and sample size were suitable for EFA (Hutcheson and Sofroniou, 1999). The EFA followed the principal axis factoring method with Promax (kappa = 4) Oblique Rotation since the factors were theorized to be correlated. The first run with 15 items produced a 3-factor unforced solution according to Kaiser’s criterion of eigenvalues greater than 1. The scree plot also indicated a 3-factor solution. These three factors accounted for 45.8, 10.99, and 5.14% of the total variance, and the overall scale explained 61.96% of the variance. Inter-factor correlations ranged from 0.48 to 0.63. Next, 6 items that had low loadings (< 0.40), loaded similarly on more than one factor, or loaded on a factor that made no theoretical sense compared to most items on that factor were deleted. With the remaining 9 items, the Bartlett’s test was statistically significant, χ^2^ = 897.320, *df* = 36, *p* < 0.001, and KMO = 0.88. This second EFA produced a clear 3-factor solution based on the eigenvalues and scree plot. The three forced factors accounted for 55, 9, and 7% of the total variance, and the overall scale explained 71% of the variance.

As seen in Table 1, the rotation sums of squared loadings suggested that the factors were fairly similar in importance. In addition, measurement convergent validity was demonstrated with all items loadings above 0.60. Measurement divergent validity was demonstrated with no cross-loadings of items and inter-factor positive correlations ranging from 0.60 to 0.66, which indicates that the factors were interrelated as theorized. As shown in the left column in Table 1, the communalities (i.e., the total amount of variance an item shares with all the other items in the analysis) ranged from 0.54 to 0.86, suggesting that each item was at least moderately and in some cases strongly related to the set of factors.

**TABLE 1:** Factor analysis results for the creative adaptability scale.

Items	Factors			
	
	Bhv.CA	Cog.CA	Emo.CA	Communality
**Bhv.CA:** When in a stressful situation, I adopt new **behaviors** that help me through it.	0.93			0.86
**Bhv.CA:** I **behave** in ways that are new to me to better deal with a stressful situation I am in.	0.86			0.80
**Bhv.CA:** I **act** in new ways to adapt to a stressful situation I am in.	0.78			0.74
**Cog.CA:** To overcome a stressful situation, I **think** of it from new perspectives		0.95		0.84
**Cog.CA:** When in a stressful situation, I **think** of it in a new way to better deal with it		0.76		0.64
**Cog.CA:** I come up with a number of original **ideas** to effectively deal with a stressful situation		0.66		0.54
**Emo.CA:** I generate new and more helpful **emotions** for dealing with a stressful situation.			0.81	0.70
**Emo.CA:** I respond **emotionally** in ways that are new to me to better tackle a problem.			0.78	0.60
**Emo.CA:** I adopt a new **emotional** response to better deal with a stressful situation.			0.71	0.66
Rotation sum of squared loadings	4.08	3.70	3.79	
	Factor correlations			
	–			
	0.60	–		
	0.66	0.60	–	

**CA, creative adaptability.**

Based on item content in relation to the theoretical basis of the measurement, the first factor (3 items) was labeled *behavioral CA* (Bhv.CA) with positive inter-item correlations ranging from 0.76 to 0.83. The second factor (3 items) was labeled *cognitive CA* (Cog.CA), with positive inter-item correlations ranging from 0.58 to 0.73. The third factor (3 items) was labeled *emotional CA* (Emo.CA) with positive inter-item correlations ranging from 0.62 to 0.67. Last, the 3-factor solution was also supported by parallel analysis which indicated that the eigenvalues extracted from this sample were larger than the eigenvalues from randomly generated correlation matrices (Patil et al., 2008, 2017). Thus, the EFA provided support for the three hypothesized interrelated dimensions underlying the CA construct. Note that although the questionnaire was originally written in Hebrew, the items listed in Table 1 were translated by the author separately and another researcher who have backgrounds in English and psychology, and back-translation was conducted to ensure the accuracy of the translation.

##### Confirmatory factor analysis

To confirm the measurement model, a CFA was performed with the cross-validation sample (*n* = 155). Modification indices for error correlations that statistically significantly improved the model’s fit were included.

The goodness-of-fit indices for the 3-factor model were *χ*^2^ = 56.349, *df* = 23, *χ*^2^/*df* = 2.45 (*p* < 0.01), TLI = 0.94, CFI = 0.96, RMSEA = 0.097, SRMR = 0.034. The factor loadings were large in magnitude, ranging from 0.68 to 0.94, indicating that all the items converged meaningfully. The same goodness-of-fit indices were detected for the 2nd-order model. Slightly better goodness-of-fit indices were obtained for a single-factor model: χ^2^ = 36.250, *df* = 21, χ^2^/*df* = 1.73 (*p* < 0.05), TLI = 0.97, CFI = 0.98, RMSEA = 0.067, SRMR = 0.033. Overall, the results indicated that all three models demonstrated good fit to the data and that CA could also be measured as a more parsimonious total score that reflects the latent CA construct underlying the theorized interrelated cognitive–behavioral–emotional ability to respond creatively and adaptively to stressful situations (the three CFA models can be found in Supplementary Material).

Multigroup CFA tested the measurement invariance across gender with the test data for the initial 3-factor model. Two models were constructed for comparison: an unconstrained model that posited a distinctive model for each gender group and a fully constrained model that posited equality (i.e., invariance in parameter estimates) between the gender groups (Byrne, 2016). The results indicated that the constrained and unconstrained models did *not* significantly differ in measurement weights (*p* = 0.21), structural covariances (*p* = 0.34), or measurement residuals (*p* = 0.30). Thus, the model was *not* significantly different for males (*n* = 73) and females (*n* = 82). Similarly, analysis with the entire sample of *N* = 310 indicated that the constrained and unconstrained models did *not* significantly differ for males (*n* = 151) and females (*n* = 159) in measurement weights (*p* = 0.46), structural covariances (*p* = 0.60), or measurement residuals (*p* = 0.75).

##### Reliability analyses and descriptive statistics

The means and standard deviations were computed for the parsimonious total score of CA for the entire sample (*N* = 310). Normality was assessed in several ways: evaluation of skewness and kurtosis values, and visual inspection of histograms, stem and leaf plots, box plots, and Q–Q plots. None of the variables showed any substantial amount of skewness or kurtosis (all < | 2|), and the data did not show a substantial departure from normality (Kim, 2013). As can be seen in Table 2, all the variables had strong internal consistency reliability, with Cronbach’s alpha values above 0.76 (McCrae et al., 2011).

**TABLE 2:** Descriptive statistics and reliability analyses for study 1.

Variable	Mean	*SD*	Skewness	Kurtosis	Cronbach’s alpha
Total CA	3.06	0.73	−0.19	0.13	0.90
Creative self-efficacy	3.73	0.68	−0.45	0.79	0.87
Openness to experience	3.42	0.54	−0.10	0.44	0.76
Spontaneity	3.09	0.90	0.06	−0.53	0.93
Well-being	3.20	0.69	0.05	−0.09	0.80
Affected by Coronavirus	3.43	1.01	−0.23	−0.43	–
Concern about Coronavirus	3.31	0.94	−0.04	−0.23	–

**N* = 310. *CA, creative adaptability.**

##### Differences by demographics

The preliminary analysis also included an examination of differences based on demographics. Age was significantly correlated with well-being (*r* = 0.17, *p* < 0.01). For gender, there was a significant difference in the well-being scores between females (*M* = 3.09, *SD* = 0.72) and males (*M* = 3.32, *SD* = 0.65), *t*(308) = 2.90, *p* = 0.004, with a medium effect size: Cohen’s *d* = 0.34. Similarly, there was a significant difference in concern about Coronavirus in females (*M* = 3.45, *SD* = 0.89) as compared to males (*M* = 3.17, *SD* = 0.97), *t*(308) = −2.66, *p* = 0.008, with a medium effect size: Cohen’s *d* = 0.30. Finally, there was a significant difference in CSE according to area of residence, with those living in the Sharon plain area of Israel reporting less CSE than those in the north, [*F*_(4, 305)_ = 3.52, *p* = 0.008], but with a very small effect size: η^2^*_*p*_* = 0.04.

#### Inter-Variable Correlations

To examine the hypothesized correlations between variables, Pearson’s r correlation coefficients were computed. As can be seen in Table 3, the CA total score correlated with CSE, openness to experience, and spontaneity, confirming Hypothesis 1. The CA total score correlated with well-being, confirming Hypothesis 2. Note that as expected, being affected by and concerned about the Coronavirus pandemic correlated negatively with well-being.

**TABLE 3:** Pearson’s correlations for all variables.

	1	2	3	4	5	6	7
Total CA	–						
Creative self-efficacy	0.39***	–					
Openness to experience	0.38***	0.58***	–				
Spontaneity	0.14*	0.15**	0.06	–			
Well-being	0.19***	0.30***	0.21***	0.10	–		
Affected by Coronavirus	−0.05	−0.10	−0.01	−0.08	-0.24***	–	
Concern about Coronavirus	0.04	0.06	0.09	−0.0	−0.26***	0.43***	–

*N* = 310. *p < 0.05, **p < 0.01, ***p < 0.001.

#### Mediation Analysis

**FIGURE 1:**
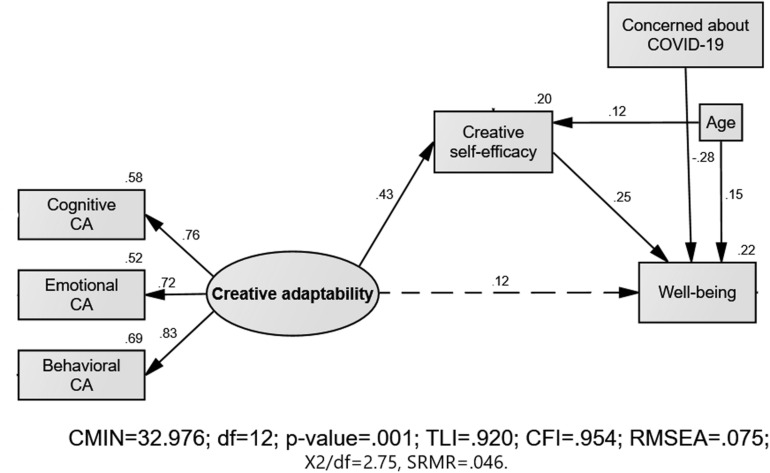
Theorized mediation model. Standardized regression weights and multiple squared correlations are presented. Solid lines indicate path coefficients that are significantly different from zero, and the dashed path indicates non-significant path coefficient.

SEM was used to test the theorized mediation model (Figure 1) where the association between CA as a latent variable and well-being was posited to be mediated by CSE, while controlling for age and Coronavirus concern. The analysis confirmed that the association between CA and well-being was fully mediated by CSE (95% CI [0.047, 0.177], *p* = 0.001 with an indirect effect of β = 0.11, *SE* = 0.03 and a total effect of β = 0.23., *SE* = 0.07). Because the CI values did not include zero, the indirect effect was significantly different from zero and the null hypothesis of no mediation could be rejected. The direct (dashed) path between CA and well-being was not significant (*p* = 0.058), thus indicating full mediation. Overall, the *R*^2^-value in Figure 1 indicated that about 22% of the variance in well-being was explained by the model, with a medium effect size of *f*^2^ = 0.30 (Cohen, 1988). Note that Coronavirus concern had a significant negative effect on well-being (β = −0.28), and overall, the analysis yielded very good fit indices, confirming Hypothesis 3. Given the cross-sectional nature of the data, and general recommendations to examine alternative mediation models with reverse causation (Hayes, 2018), an alternative model was tested where the association between CSE and well-being was posited to be mediated by CA as a latent variable. However, this reverse mediation model failed to yield a significant mediation, 95% CI [−0.015, 0.136], *p* = 0.13.

#### Moderation Analysis

Protective factors are theoretically “moderators or buffers of the impact of exposure to risk, operationalized as significant interactions of the protective factors with the risk factors in regression analyses” (Jessor and Turbin, 2014, p. 1039). It was theorized that CA, as a protective factor, would moderate the effect of participants’ concern about the Coronavirus on their well-being. Specifically, it was hypothesized that an increase in CA (moderator) would decrease the effect of the Coronavirus concern (predictor) on well-being (outcome), with age, gender, and affected-by-Coronavirus as covariates.

**FIGURE 2:**
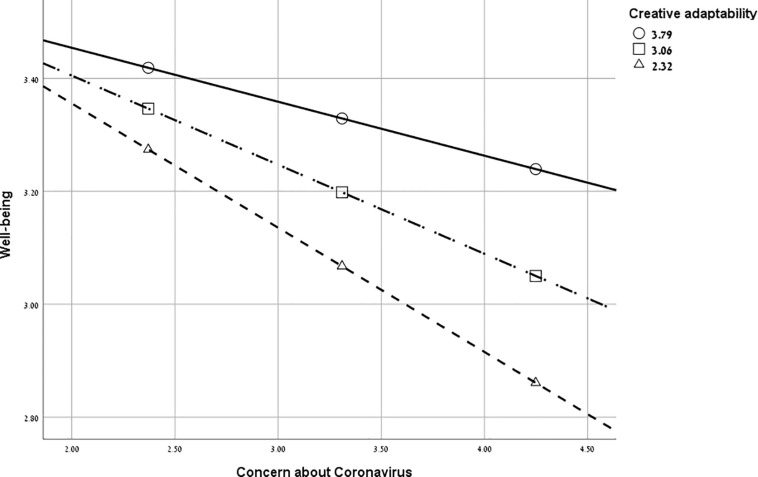
Moderation analysis. Creative adaptability moderates the effect of concern about Coronavirus on well-being.

A model with the CA total score as a moderator was computed. The interaction term was statistically significant, confirming Hypothesis 4. As shown in Table 4, concern about Coronavirus significantly related to less well-being, and CA significantly moderated this relationship, as illustrated in Figure 2. The interaction was probed by testing the conditional effects of concern about Coronavirus at three levels of CA, one standard deviation below the mean, at the mean, and one standard deviation above the mean. As shown in Table 4, concern about Coronavirus was significantly related to well-being when CA was one standard deviation below the mean and when at the mean (*p* < 0.001), but not when CA was one standard deviation above the mean (*p* = 0.08). The Johnson–Neyman technique showed that the relationship between concern about Coronavirus and well-being was significant when CA was less than 3.7 standard deviations above the mean but not significant with higher values of CA. This hints that CA may buffer the impact of individuals’ concern about Coronavirus on their well-being.

**TABLE 4:** Concern about Coronavirus predicting well-being moderated by creative adaptability.

	B	*p*	95% CI	
Concern about Coronavirus	−0.42	0.003	−0.688	−0.146
Creative adaptability	−0.10	0.51	−0.405	0.200
Interaction ^*a*^	0.085	0.05	0.000	0.170
Age	0.007	0.004	0.002	0.011
Gender	−0.11	0.14	−0.262	0.038
Affected by Coronavirus	−0.08	0.052	−157	0.001

[*F*_(6,303)_ = 10.33, *p* < 0.001], *R* = 0.41, *R*^2^ = 17.				

**Conditional effect of Coronavirus concern on well-being**				

**Creative adaptability**	**B**	***p***	**95% CI**	

−1*SD* (2.32)	−0.22	< 0.001	−0.325	−0.115
Mean (3.06)	−0.16	< 0.001	−0.245	−0.072
+ 1*SD* (3.79)	−0.09	0.08	−0.202	0.011

**N = 310. a, interaction of concern about Coronavirus with creative adaptability.**

### Study 1 Summary

Preliminary analyses in Study 1 involved the initial testing and refinement of the CA scale. While EFA results provided support for the three hypothesized CA dimensions, subsequent CFA indicated that the 9-item CA could also be measured as a latent construct underlying these three interrelated factors. Given these results, and the relatively high correlations between the three factors, a parsimonious total score was used in further analyses. The CA correlations with other measures (CSE, openness to experience, spontaneity) indicated the scale’s divergent validity, and the Cronbach’s alpha demonstrated strong internal consistency reliability. SEM analysis confirmed the theorized mediation model where the association between CA and well-being was mediated by CSE. Finally, moderation analysis confirmed that CA may buffer the impact of individuals’ concern about Coronavirus on their well-being. These results are explored further in the General Discussion below.

## Study 2

Using a 2-wave short-term longitudinal design, Study 2 was designed to (1) examine whether CA would predict lower psychological stress levels across a 2 week interval when Coronavirus was on the rise and the lockdown restrictions were made stricter, and to (2) examine the test–retest reliability of the CA scale.

### Materials and Methods

#### Sample and Procedure

In April 2020, students in psychology courses participated in the study for research participation credit. At Time 1, 77 students responded (aged 18–40, *M* = 34, *SD* = 4.4), of whom 94% were female; 92% were born in Israel and the rest in “other” countries. Of the sample, 95% lived in the north of Israel where the university is located. Regarding religion, 73% were Jewish, 13% were Muslim, 1% Christians, and the rest “other.” In terms of level of religiosity 60% were secular, 30% traditional, and 10% observant. Regarding marital status, 58% were single, 36% were married or living with a partner, 4% were divorced or separated, and 2% were “other.” Most participants (94%) did not have children and 65% reported their financial situation to be “average,” 31% reported “below average,” and 3% “above average.”

Of the 77 students from Time 1, 71 responded at Time 2, with sample attrition of six students (7.8%). Logging in to the survey platform (Qualtrics) indicated consent and since all questions were mandatory, there were no missing data. Study 2 received the same ethical approval as Study 1.

#### Measures

The same 9-item CA scale and demographics questionnaire used in Study 1 were administered in Study 2. In addition, the 4-item Brief Stress Scale was administered with reference to “over the last month” (Cohen et al., 1983). Items were rated on a scale from 1 (*never*) to 5 (*always*). A sample item is: “In the last month, how often have you felt that you were unable to control the important things in your life?”

#### Data Analysis

Normality was assessed in the same way as in Study 1. To examine whether the Time 1 CA would predict lower psychological stress levels 2 weeks later during the Coronavirus surge, a hierarchical multiple-regression analysis was computed with Time 2 psychological stress as the dependent variable. To examine test–retest reliability for the total CA score at Time 1 and Time 2, intra-class correlation coefficients (ICC) and their 95% confident intervals (CI) were calculated using SPSS v25, based on absolute agreement and a 2-way mixed-effects model (Koo and Li, 2016).

### Study 2 Results

None of the variables showed any substantial amount of skewness or kurtosis and the data did not show a substantial departure from normality (Kim, 2013). As can be seen in Table 5, all the variables had good internal consistency reliability with all Cronbach’s alpha values above 0.75 (McCrae et al., 2011). There were no differences in CA and psychological stress for any of the demographic variables.

**TABLE 5:** Study 2 descriptive statistics and reliability analyses.

Variable	*Mean*	*SD*	Skewness	Kurtosis	Cronbach’s alpha
T1 Total CA	2.93	0.72	−0.38	0.28	0.89
T1 Psychological stress	2.85	0.79	−0.16	−0.25	0.82
T1 Affected by Coronavirus	3.25	0.89	−0.28	−0.20	–
T1 Concern about Coronavirus	3.44	0.82	−0.25	0.21	–
T2 Total CA	3.09	0.60	−0.35	1.61	0.88
T2 Psychological stress	3.45	0.29	0.37	0.31	0.75

**T1 N, 77; T2 N, 71. CA, creative adaptability.**

#### Prediction of Psychological Stress

Hierarchical multiple-regression analysis was computed to test whether CA at Time 1 would predict lower psychological stress at Time 2, when controlling for participants’ age and concern about the Coronavirus from Time 1. In the first step, psychological stress was regressed on age and concern about the Coronavirus using the enter method [*F*_(1,69)_ = 4.06, *p* = 0.048]. In the second step, the CA total score was added to the regression using the enter method [*F*_(2,68)_ = 5.71, *p* = 0.005]. The tolerance value exceeded.10 and variance inflation factors (VIF) were less than 10, indicating no problems with multicollinearity between the independent variables (Meyers et al., 2013). As can be seen in Table 6, concern about the Coronavirus at Time 1 predicted psychological stress at Time 2 (β = 0.24) and CA in Time 1 predicted lower psychological stress at Time 2 (β = −0.30), 2 weeks later. CA contributed 8.8% to the variance accounted for by the model, [*F*_(1,68)_ = 7.01, *p* = 0.01]. Overall, the regression model explained 14% of the variance in psychological stress.

**TABLE 6:** Study 2 hierarchical regression analysis summary for creative adaptability predicting psychological stress.

	*B*	*SE B*	β	*R*^2^	Δ*R*^2^
Step 1:				0.056	0.056*
T1 Concern about Coronavirus	0.19	0.09	0.24		
Step 2:				0.144	
T2 Creative adaptability	−0.27	0.10	−0.30		0.088**

T1 = time 1, T2 = time 2. N = 71. *p = 0.05, **p = 0.01.

#### Test–Retest Reliability

Test–retest reliability was examined over an interval of 2 weeks (April 2020). ICC was computed with a 2-way mixed-effects model with absolute agreement. The obtained test–retest reliability coefficient was 0.71 [95% CI (0.493, 0.813)], indicating moderate to good reliability for the scale scores over the 2 week interval (Koo and Li, 2016).

### Study 2 Summary

Results from the short-term longitudinal data in Study 2 lend further weight to the cross-sectional results in Study 1, indicating that CA may predict positive outcomes. Specifically, in Study 2, CA predicted lower psychological stress across a 2 week interval at a time when the Coronavirus was spreading and the lockdown restrictions were made stricter across Israel. The findings provide further support for the CA scale’s internal consistency reliability and contribute to establishing its test–retest reliability, which reflects temporal stability.

## General Discussion

The purpose of this article was to introduce CA, its framework, measurement, correlates, and outcomes. Data were gathered from two samples of adults during the 2020 outbreak of the COVID-19 pandemic in Israel. The results of preliminary EFA and CFA in Study 1 supported the 9-item CA scale measurement model, in terms of the theorized underlying construct and its interrelated dimensions of cognitive, behavioral, and emotional CA. Given the relatively high correlations between the three factors, some caution should be exercised when using the three separate subscales, which may cause collinearity issues when attempting to use the three variables as predictors in a multivariate regression model (Pituch and Stevens, 2016). Researchers are therefore advised to use the 9-item single total score that was also deemed reliable, theoretically more complete (i.e., integrative) and operationally more parsimonious. Initial evidence for the divergent validity, internal consistency reliability, and predictive validity of the CA scale total core were also obtained.

The results also provide evidence supporting the hypothesized positive correlations between CA and spontaneity, consistent with Moreno’s claim that spontaneity and creativity are closely related (Moreno, 1955). Similarly, based on the positive correlation between CA and openness, as well as theoretical reasoning, it could be argued that people’s CA linked to their openness and ability to generate new ideas, experience and express new emotions, and enact new behaviors to handle the demands of a stressful situation (Ferguson and Bibby, 2012).

Participants in this study experienced the outbreak of the COVID-19 pandemic, which has been associated with numerous mental health problems, including increased stress, depression, irritability, fear, confusion, anger, and frustration (Brooks et al., 2020). The CA’s positive correlations with well-being, its negative correlation with concern about Coronavirus, and the 2 week prediction of lower psychological stress provide initial evidence that CA may play a role as a personal protective factor. This claim is further supported by the result that CA moderated the effect of participants’ concern about the Coronavirus on their well-being. In other words, an increase in CA decreased the effect of the Coronavirus concern on well-being, thus buffering the impact of risk. Overall, the results here are congruent with the positive psychological and humanistic notion that creativity is a personal resource that can be consequential to one’s well-being (Rogers, 1961; Maslow, 1962; Peterson and Seligman, 2004).

Another important theoretical contribution of these studies is a better understanding of *how* CA relates to well-being. Specifically, the findings provide insight into the potential role of CSE as a mediator through which CA may influence well-being. It seems plausible that people’s CA may enhance their self-perceived ability to be creative (i.e., CSE) when faced with a stressful situation, which may in turn enhance their well-being. This conceptualization is consistent with the conservation of resources theory, which posits that personal resources tend to generate gains in other resources, which in turn may result in greater well-being (Hobfoll, 2002, 2011). The mediating role of CSE corroborates previous findings (Orkibi and Ram-Vlasov, 2019) and Bandura’s (1997) claim that strong efficacious beliefs enhance well-being because people who believe in their capabilities perceive difficulties as challenges to be mastered rather than as threats to be avoided. As noted above, the reverse mediation model (with CSE as a predictor) was not significant. Finally, from a developmental perspective (Barbot and Heuser, 2017), it is worth noting that CA may play a role in the formation of a positive self-definition and identity. Specifically, successful CA (i.e., that maximizes positive outcomes and minimizes negative outcomes) may affirm a person’s positive sense of self. However, the causal directionality of this claim warrants further examination because it is also plausible that a positive sense of self may lead to better CA, or that a feedback loop may exist.

### Study Limitations

While the present studies provide initial support for the protective role of CA, at least three potential limitations should be recognized and addressed in future research. First, the cross-sectional data in Study 1 and the 2-wave short-term longitudinal design in Study 2 limit causal inferences. The temporal relationships between individuals’ exposure to risk (i.e., COVID-19), their CA and well-being outcomes warrant further examination. Future studies should collect data over a longer period of time to monitor changes in CA and well-being indicators. Second, all the measurements in this study were self-reported, which may be susceptible to social desirability bias, although there is evidence that self-report measures are far less problematic than some have assumed (Silvia et al., 2012). Moreover, it is reasonable to measure self-perceptions and subjective experiences (thoughts and emotions) with self-reports, because a perceived need for adaptation depends on subjective personal interpretations of a situation (Runco, 1999). That is, individuals who experience the same event can be affected differently, so that while one person may see a problem, or interpret a situation as stressful, another may not. Still, future studies may use additional sources of data to examine the extent to which a person’s CA is corroborated by others, such as a spouse or colleague. Finally, a larger sample with a more balanced gender distribution would increase the generalizability of the results and hence the applicability of the CA construct.

### Implications and Future Directions

Despite these possible limitations, these studies have several practical implications. Drawing on previous studies on creativity interventions (Vally et al., 2019; Sun et al., 2020), and improvisational theater training (Felsman et al., 2020), it is conceivable that CA may be enhanced through intentional practice. This idea is also consistent with traditional cognitive–behavioral therapy approaches that focus on altering unhelpful thoughts, emotions, and behaviors (Beck, 2011) as well as newer approaches, such as acceptance and commitment therapy (Hayes et al., 2012), which aim to increase clients’ psychological flexibility so that they can better respond to change. In particular, psychodrama and drama therapy interventions, which involve creative and expressive exploration of thoughts and feelings, as well as behavioral role-play in real or imagined situations (Azoulay and Orkibi, 2015; Orkibi and Feniger-Schaal, 2019; Feniger-Schaal and Orkibi, 2020), may potentially enhance clients’ ability to respond to changes. In light of the three interrelated dimensions of CA, an intervention program could consist of the exploration of more adaptive cognitive appraisals and solutions, self-regulation of emotions to generate more adaptive emotions, and behavioral modifications to act in a way that is better adapted to the demands of the situation. For example, an intervention for either prevention or treatment may rely on the positive psychodrama framework (Orkibi, 2019) to draw on the value of CA as a personal resource. Specifically, the double technique (in which the therapist or a group member offers a client alternative cognitions or emotions) may help clients come up with new and more adaptive thoughts (e.g., interpretations) or feelings in response to a changed or stressful situation. The role reversal technique (in which a client takes on the role of another person) promotes perspective taking and empathy which may also help replace old maladaptive thoughts and feelings with new adaptive ones. The mirror technique (in which a client watches someone else replaying what s/he previously enacted) and the future projection technique (in which clients project themselves into the future as if it were in the here-and-now) may promote behavioral alteration by allowing clients to explore new and more adaptive behavioral responses. This creative–expressive work may strengthen clients’ belief in their ability to creatively and adaptively respond to a stressful situation, which may in turn enhance their well-being.

Thus overall, the present study shows the potential of the CA construct and its relationship with well-being. It is hoped that this current research will stimulate further investigation of CA as a protective factor not only for global crises but also for everyday challenges.

## Data Availability Statement

The datasets presented in this article are not readily available because the dataset is part of a larger project. Requests to access the datasets should be directed to HO, horkibi@univ. haifa.ac.il.

## Ethics Statement

The studies involving human participants were reviewed and approved by the Ethics Committee for Human Experiments, Faculty of Social Welfare and Health Sciences, University of Haifa (#397/16-1). The patients/participants provided their written informed consent to participate in this study.

## Author Contributions

HO was the only contributor to this publication.

## Conflict of Interest

The author declares that the research was conducted in the absence of any commercial or financial relationships that could be construed as a potential conflict of interest.
