# Supplementary material for: Creative Adaptability: Conceptual Framework, Measurement, and Outcomes in Times of Crisis
Source: Front Psychol. 2021 Jan 12;11:588172. doi: 10.3389/fpsyg.2020.588172 (PMC7835130; doi:10.3389/fpsyg.2020.588172)
Supplement: Supplementary file 1 [file Data_Sheet_1.PDF]

Supplementary Material

Creative Adaptability:

Conceptual Framework, Measurement, and Outcomes in Times of Crisis

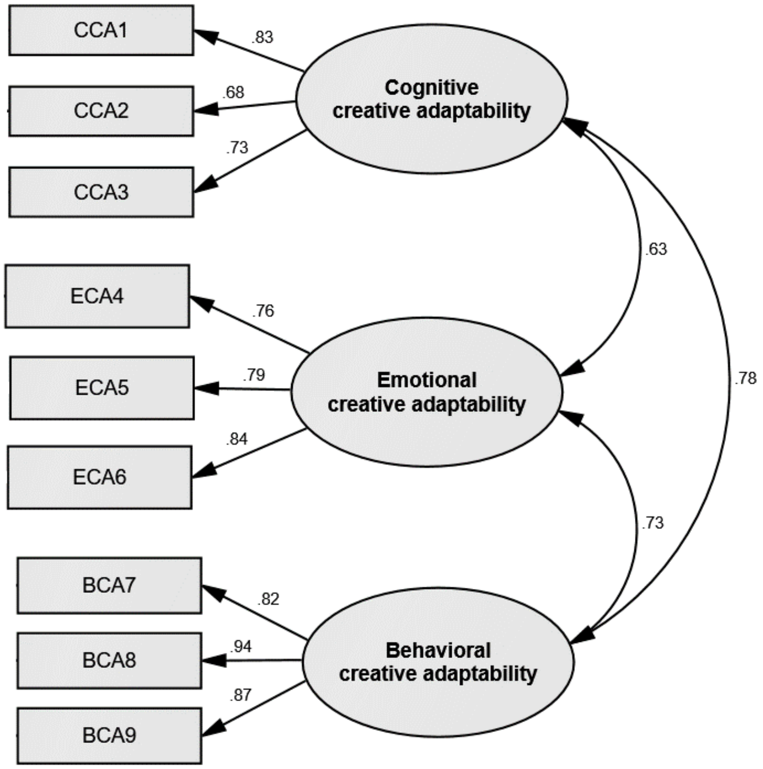

CMIN=56.349; df=23; p-value=.000; TLI=.942; CFI=.963; RMSEA=.097;

Figure S1. CFA for the 3-Factor Creative Adaptability Model

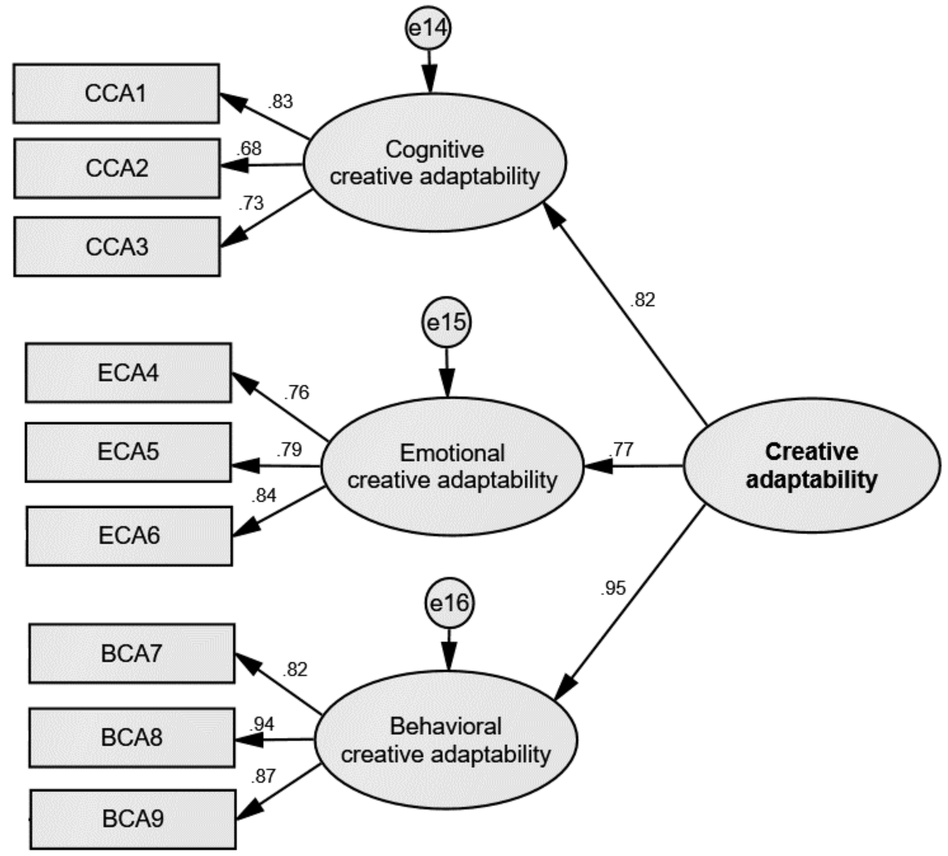

CMIN=56.349; df=23; p-value=.000; TLI=.942; CFI=.963; RMSEA=.097;

Figure S2. CFA for the Second-Order Creative Adaptability Model

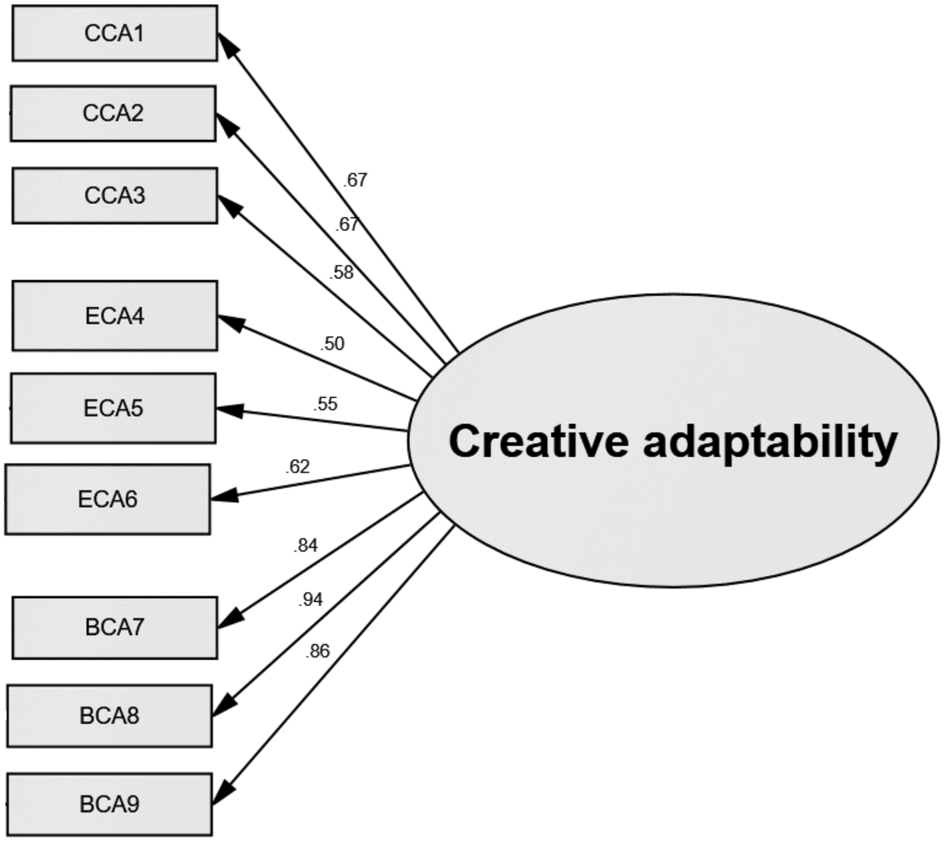

CMIN=36.250; df=21; p-value=.020; TLI=.971; CFI=.983; RMSEA=.069;

Figure S3. CFA for the 1-Factor Creative Adaptability Model
